# Response to Stampfer Commentary

**DOI:** 10.1371/journal.pmed.0020311

**Published:** 2005-09-27

**Authors:** David F Williamson

**Affiliations:** **1**Centers for Disease Control and PreventionAtlanta, GeorgiaUnited States of America

Stampfer's recent Perspective [1] on the paper by Sørensen et al. [2] appropriately acknowledges the challenges inherent in using observational epidemiology to determine the impact of weight loss on life expectancy. However, his case that the data of Sørensen et al. do not support their conclusion that intentional weight loss may be hazardous is based, in part, on erroneous statements about the study.

Stampfer suggests that “reverse causation” could account for the findings of Sørensen et al. because he believes they did not do a “lagged” analysis in which deaths that occur in the first few years after follow-up are excluded. In the statistical analysis, however, Sørensen et al. describe using two separate fully adjusted models: one for the first five years of follow-up and one for the period thereafter, and they also reported mortality hazard ratios (HRs) associated with intentional weight loss during each period. Because so few deaths occurred in the first five years of follow-up, the estimated mortality HR for intentional weight loss during this period (6.26) had such a wide confidence interval (0.33–118) that it was essentially meaningless. However, after excluding the first five years of follow-up data, Sørensen et al. still found a clinically and statistically significant association between intentional weight loss and death during the remaining 13 years of follow-up: HR = 1.88 (confidence interval, 1.05–3.39).

Stampfer indicates that the authors differentiated only between current smokers and nonsmokers and, thus, inappropriately combined never smokers with past smokers. In their methods, however, Sørensen et al. reported that they originally used four categories (never smoker, occasional smoker, former regular smoker, and current smoker) to code the smoking status of the study's participants, before recoding smoking status as a dichotomous yes-or-no variable. However, as Sørensen et al. described in their statistical analysis, they analyzed their models using both of the coding methods to determine whether recoding resulted in residual confounding. Because they found no residual confounding, they chose to report results only from the model with the simpler, dichotomous coding of smoking status.

Stampfer also argues that the best way to remove residual confounding by smoking is to “simply exclude current and past smokers” [1]. This exclusionary approach for smoking has been previously examined in a methodological study that utilized statistical simulation, with data from 15 diverse observational studies of body weight and mortality [3]. The study concluded that eliminating smokers from the datasets prior to analysis produces results similar to those expected from the elimination of numerically similar random proportions of the datasets prior to analysis [3]. Thus, the practice of excluding smokers in studies of weight loss and mortality is highly questionable.

## References

[pmed-0020311-b1] Stampfer M (2005). Weight loss and mortality: What does the evidence show?. PLoS Med.

[pmed-0020311-b2] Sørensen TIA, Rissanen A, Korkeila M, Kaprio J (2005). Intention to lose weight, weight changes, and 18-y mortality in overweight individuals without comorbidities. PLoS Med.

[pmed-0020311-b3] BMI in Diverse Populations Collaborative Group (1999). Effect of smoking on the body mass index–mortality relation: Empirical evidence from 15 studies. Am J Epidemiol.

